# Supplementary material for: The Effect of Breast Milk Microbiota on the Composition of Infant Gut Microbiota: A Cohort Study
Source: Nutrients. 2022 Dec 19;14(24):5397. doi: 10.3390/nu14245397 (PMC9781472; doi:10.3390/nu14245397)
Supplement: Supplementary file 1 [file nutrients-14-05397-s001.zip › nutrients-2049970-supplementary.pdf]

Supplementary

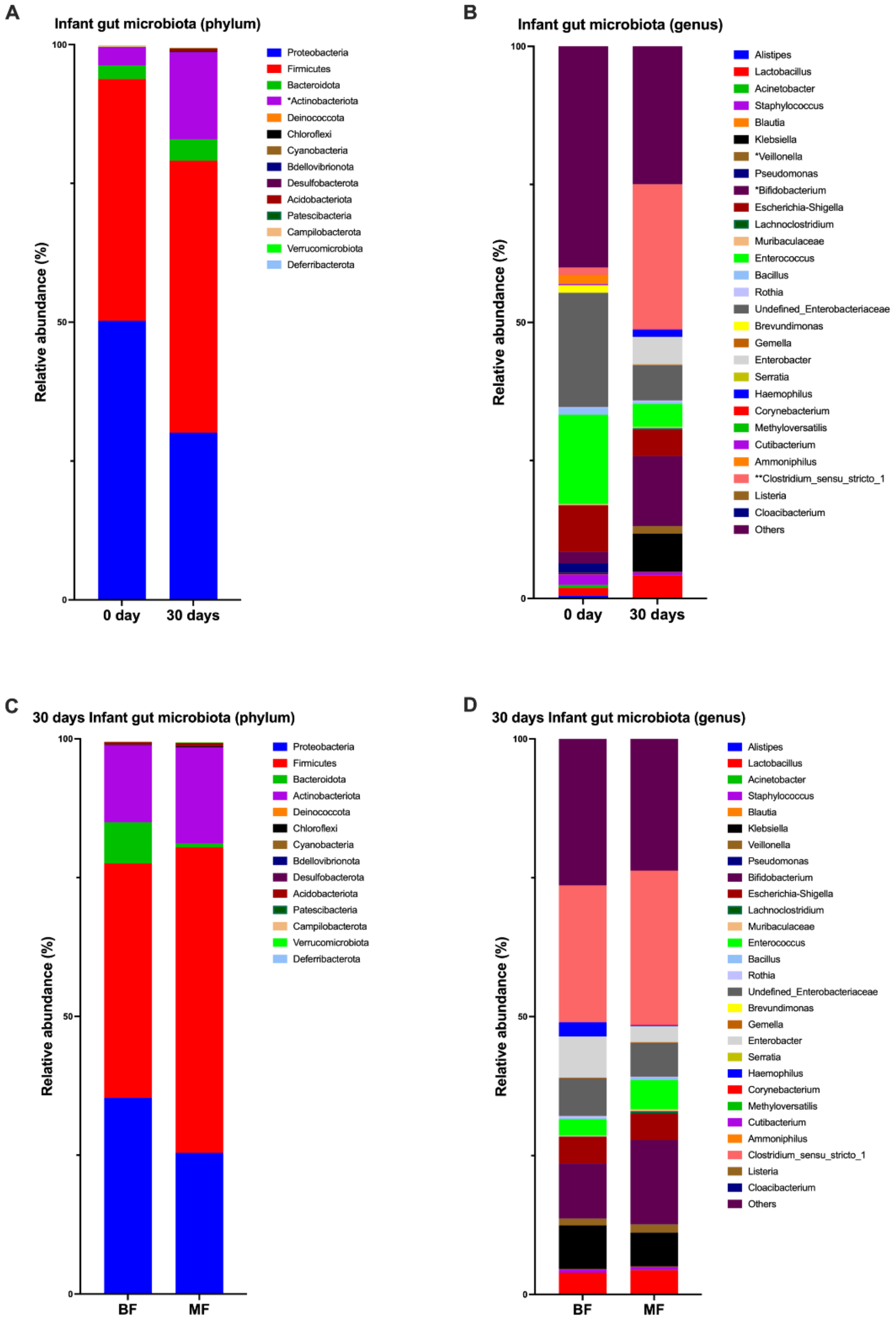

Figure S1. Differences in the relative abundance of infant gut microbiota among different periods and subgroups.（0d, n = 6; 30d, n = 19; BF, n = 9; MF, n = 10）(A) Differences in the relative abundance of infant gut microbiota at 0 d and 30 days at the phylum level; (B) Differences in the relative abundance of infant gut microbiota at 0 day and 30 days at the genus level; (C) Differences in the relative abundance of infant gut microbiota at the phylum level on 30 days; (D) Differences in the relative abundance of infant gut microbiota at the genus level on 30days. * p<0.05， ** p<0.01

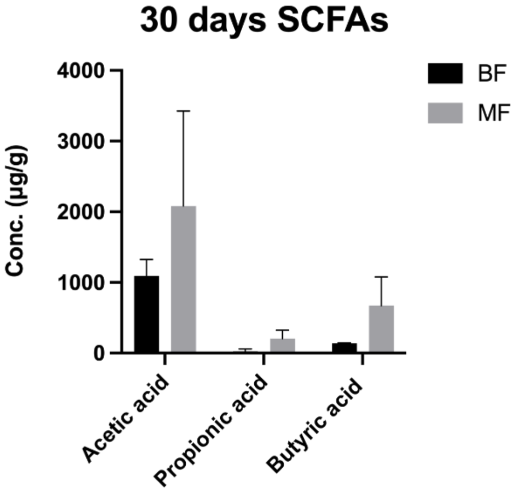

Figure S2. The concentrations of SCFAs on 30 days between BF and MF.（BF, n = 9; MF, n = 10）.
